# Retrospective analysis of 426 donors of a convalescent collective after mild COVID-19

**DOI:** 10.1371/journal.pone.0247665

**Published:** 2021-02-23

**Authors:** Tobias Flieder, Tanja Vollmer, Benjamin Müller, Jens Dreier, Bastian Fischer, Cornelius Knabbe, Ingvild Birschmann

**Affiliations:** Herz- und Diabeteszentrum Nordrhein-Westfalen, Universitätsklinik der Ruhr-Universität Bochum, Institut für Laboratoriums- und Transfusionsmedizin, Bad Oeynhausen, Germany; Institut Cochin, FRANCE

## Abstract

**Background:**

The novel coronavirus disease 2019 (COVID-19), caused by the severe acute respiratory syndrome coronavirus 2 (SARS-CoV-2), has spread across the world. The aim of our study was to characterize mild courses and to determine the antibody status for these patients.

**Methods:**

We initiated an appeal for convalescent plasma donations. 615 people contacted us, and we ultimately included 426 in our analyses, in whom it was possible to assume COVID-19 based on detection of specific SARS-CoV-2 antibodies or virus detection during the disease using RT-PCR.

**Results:**

The median duration of the disease was 12 days and the most common symptoms were fatigue, cough and olfactory and gustatory dysfunction. Anti-SARS-CoV-2 IgG was detected in 82.4% of the persons and IgA antibodies were found in 73.9%. In 10.8%, no antibodies were detectable despite a positive RT-PCR result during the disease. Nevertheless, of 24 persons with asymptomatic courses of COVID-19, antibodies against SARS-CoV-2 could be detected in 23 (96%). Furthermore, there was a correlation between the duration of the disease and the detection of IgG antibodies. In addition, a correlation between the determined IgG antibodies and neutralizing antibodies was shown.

**Conclusion:**

In this study, we were able to describe mild COVID-19 courses and determine antibody statuses for them. It could be shown that, despite SARS-CoV-2 detection during the disease, not all individuals developed antibodies or their level of antibodies had dropped below the detection limit shortly after the end of the disease. The extent to which immunity to re-infection is given in persons with undetectable antibodies (IgG, IgA) needs to be investigated in future studies.

## Introduction

The worldwide pandemic of severe acute respiratory syndrome coronavirus 2 (SARS-CoV-2), which causes coronavirus disease 2019 (COVID-19), is a novel disease for which there is no data sufficient for long-term management. This includes virulence, the factors that determine the course of the disease, as well as the immunity situation in the many varied forms. The spread of the virus was characterized as a pandemic by the World Health Organization (WHO) on March 11^th^ 2020. At the present time (January 27^th^, 2021), nearly 100 million people have been infected with the virus. The symptoms of COVID-19 are unspecific. At the beginning of the pandemic, the occurrence of fever, cough and fatigue was frequently reported [1, 2]. In the course of the pandemic, other frequently occurring symptoms were also identified, such as loss of the sense of smell and taste [3, 4].

Currently, there exists no specific drug for the treatment of COVID-19. Drugs like Remdesivir or Dexamethasone demonstrated promising results in some recently published studies, but their benefit, especially for the outcome of the patients, has to be proven in further investigations [5–7]. An important step in managing the pandemic is the approval of the first vaccines in late 2020/early 2021, which show high efficacy [8, 9].

Another form of therapy, which has already been successfully used in earlier viral diseases, such as severe acute respiratory syndrome coronavirus (SARS) [10] or middle east respiratory syndrome (MERS) [11], is the transfusion of convalescent plasma (CP) from persons who have recovered from COVID-19 [12, 13]. The use of CP for the therapy of COVID-19 has been investigated in some studies and case series with different conclusions about efficacy [10, 13–16]. However, a recent double-blind, placebo-controlled trial has now shown that early administration of high-titer CP can prevent severe disease progression [17].

Further essential uses of CP are the production of a hyperimmune preparation against SARS-CoV-2 or its use for diagnostic purposes, such as control material in the context of antibody tests.

In order to obtain enough plasma for the abovementioned applications, we used various types of media to recruit volunteers who had recovered from COVID-19. Fortunately, many people felt moved to support us in this project. Due to the overwhelming willingness of the people from our region, we had a large collective available. In addition to collection of the CP, we could also gather clinical and laboratory chemical data from COVID-19 recovery volunteers.

At the beginning of the study in March, there were no effective drugs against SARS-CoV-2, which is why we received approval from our district government for the production of convalescent plasma on March 27^th^, 2020. This made us one of the first donor establishments in Germany to start producing CP. Between April 1^st^ and June 20^th^, 2020, 615 people contacted us, of whom we invited 485 potential CP donors for an initial examination.

The aim of our study was to provide a regional characterization of the CP donor collective, especially in mild cases, and to determine, for example, the relationship between disease severity and antibody expression.

## Methods

### Study design

Between April 1^st^ and June 20^th^, 2020, 615 people contacted us after overcoming what was thought to be COVID-19. 485 of these persons were invited to our clinic for an initial examination and were thereafter included in the study. Institutional Review Board approval was obtained from the ethics committee of Bad Oeynhausen (Reg.-No. 670/2020). Since this is a retrospective data analysis, no informed consent was required from the subjects. The data set was anonymized before it was analyzed. The individuals presented themselves at our clinic at least 14 days after they were symptom-free. Exceptions were symptoms such as olfactory and gustatory dysfunction and fatigue, some of which lasted several weeks, even though the other symptoms had already subsided. To record the course of the disease, all potential donors were ask to fill in a questionnaire on which they indicated when the first symptoms appeared and how long the individual symptoms lasted.

In addition, a throat swab for SARS-CoV-2 RNA detection by RT-PCR was taken and the presence of anti-SARS-CoV-2 IgG and IgA antibodies was investigated. Furthermore, all mandatory tests required for each blood donation were performed.

### Detection of anti-SARS-CoV-2 IgG and IgA

Detection of the anti-SARS-CoV-2 IgG and IgA antibodies was performed semi-quantitatively with the kits "Anti-SARS-CoV-2 ELISA (IgG)" and "Anti-SARS-CoV-2 ELISA (IgA)" from EUROIMMUN AG (Lübeck, Germany) using the Analyzer I device. The tests detect antibodies against the S1 domain of the spike protein, including the receptor binding domain. The test was performed according to the manufacturer’s instructions.

Semiquantitative results were calculated as a ratio of the extinction of samples over the extinction of a calibrator. Results of the immunoassay were classified into the three categories negative (ratio < 0.8), borderline (ratio > 0.8 to ≤ 1.1), positive (ratio > 1.1).

### Detection of anti-SARS-CoV-2 neutralization antibodies

The surrogate virus neutralization test (sVNT) from GenScript (GenScript cPass™ SARS-CoV-2 Neutralization Antibody Detection Kit, Genscript, The Netherlands) was used to measure neutralizing antibodies against SARS-CoV-2. The test was performed according to the manufacturer’s instructions. The assay contains the receptor binding domain (RBD) of the spike protein of SARS-CoV-2 coupled to a horseradish peroxidase (HRP) and the ACE2 receptor. The presence of neutralizing antibodies in the sample blocks the interaction of the RBD-HRP and the ACE2 receptor. Results are expressed as % inhibition and calculated using the negative control. A value of ≥20% is considered positive.

A total of 140 samples were measured with this test. The samples were divided into three groups depending on the results of the anti-SARS-CoV-2 ELISA (IgG). Group 1 (ratio: >1.1 - ≤2) included 46 samples, group 2 (ratio: >2 - ≤4) included 46 samples, and group 3 (ratio: >4–10) included 48 samples.

### Detection of SARS-CoV-2 with RT-PCR

For detection of the SARS-CoV-2 virus in the swab samples, two different assays were used. Extraction of total RNA was performed using the AltoStar AM16 (ADT) automated RNA/DNA extraction system according to the manufacturer’s recommendations. Amplification was carried out using the Real-Star SARS-CoV-2 RT-PCR Kit (ADT) on the CFX96 real-time thermal cycler (Bio-Rad, Feldkirchen, Germany).

Second, the fully automated cobas SARS-CoV-2 assay (Roche Diagnostics, Mannheim, Germany) on the cobas 6800 platform was used. Swab samples were processed on both systems according to the manufacturer’s instructions.

### Further laboratory parameters

Further laboratory parameters were determined using the Architect c8000 and i2000SR platform (Abbott Diagnostics, Illinois), the ADVIA 2120i (Siemens Healthcare, Erlangen) and the CS-5100 (Siemens Healthcare, Erlangen).

### Statistical analysis

All continuous data are presented as the mean ± standard deviation (SD) or median ± interquartile range (IQR). Normality testing was performed using the Shapiro–Wilk test. Categorical data are presented as numbers and percentages. Chi-square tests were conducted for analysis of the categorical variables, and t-tests were conducted for analysis of the continuous variables. For correlation determination, Spearman’s correlation coefficient was calculated since the variables did not show normal distribution.

## Results

### Baseline and demographic data

**Fig 1:**
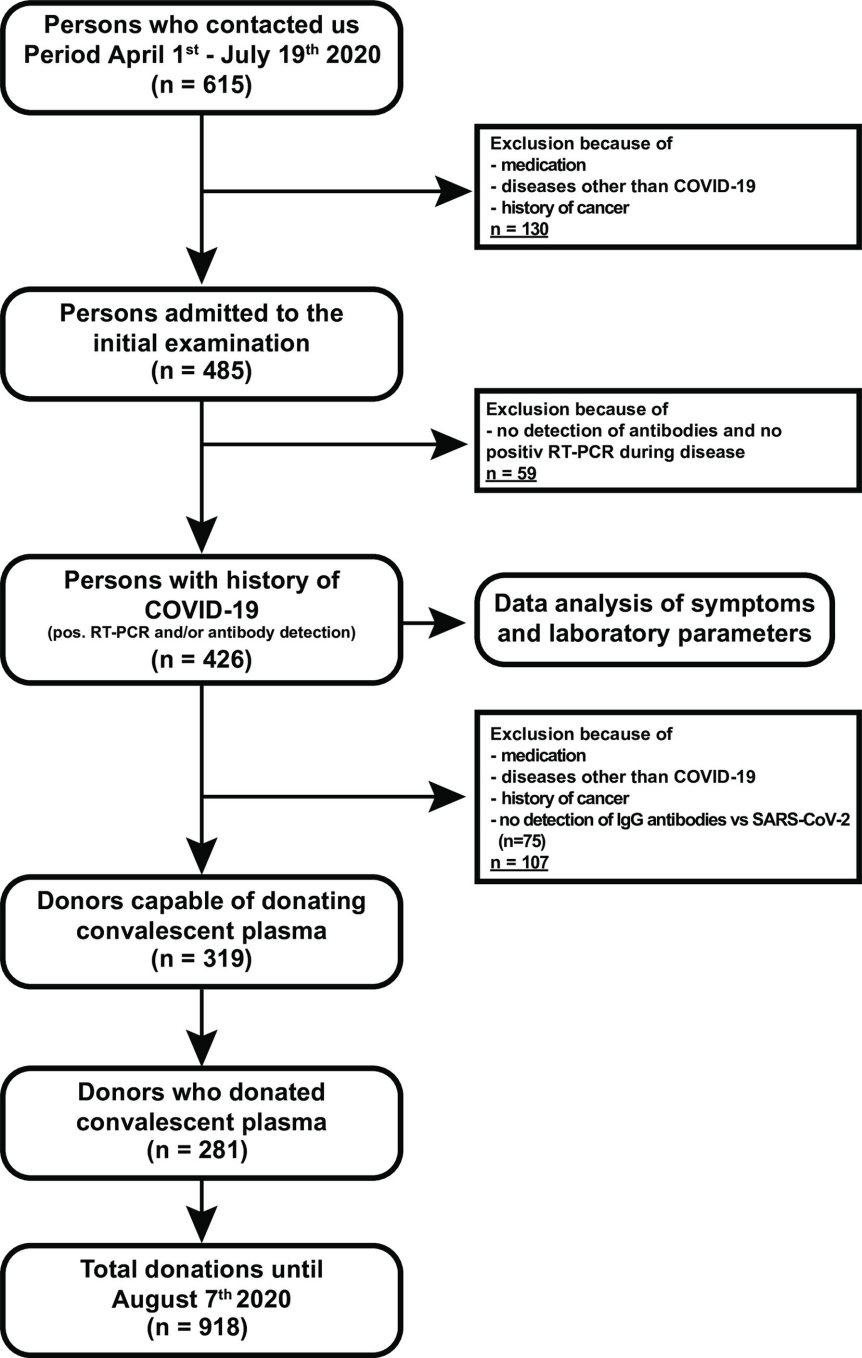
Flowchart of the study design.

In this study, 485 persons were recruited who were thought to have recovered from a mild course of COVID-19 (Fig 1). Of these individuals, 59 had to be excluded because they either had no detectable antibodies against SARS-CoV-2 or no positive RT-PCR during the disease. The remaining 426 individuals could be assumed to have had COVID-19, based on a positive RT-PCR result (throat swabs taken during the disease) and/or the detection of antibodies (IgG and/or IgA) against SARS-CoV-2, and were therefore included in our study. The median age of the donors was 47 (IQR: 34–54) years, and 50.7% were female (216/426). The subjects were included in the study with a median of 36 days after the end of the disease (Table 1). The median time between the onset of symptoms and presentation in our clinic was 50 days (IQR: 41–61) (Table 1, S1 Fig). The blood group distribution corresponds to the expected blood group distribution in central Europe, as well as to our normal donor collective (data from March 1^st^, 2016—March 1^st^, 2020), with blood group B as the only exception (Table 2). Due to the fact that IgG antibodies against SARS-CoV-2 had to be detectable for a CP donation, 75 further persons were excluded. In addition, 32 people had to be excluded because of medication or certain underlying diseases (e.g. history of cancer, chronic autoimmune disease,…). Finally, 319 volunteers were eligible to donate.

**Table 1:** Demographic and laboratory baseline date of persons after mild COVID-19. An extended set of laboratory analyses was performed for 260 persons.

	**n = 426**	**Reference value**
**Age [years]**		
Median (IQR)	47 (34–54)	
**Sex [female] n (%)**	216 (50.7)	
**Blood group n (%)**		
0	159 (37.3)	
A	214 (50.2)	
B	32 (7.5)	
AB	16 (3.8)	
NA	5 (1.2)	
**Laboratory parameters**		
Total protein [g/dL]		6.5–8
median (IQR)	7.5 (7.2–7.8)	
IgG [mg/dL]		500–1800
median (IQR)	1090 (962–1240)	
Hämoglobin [g/dL]		12.5–17
mean (sd)	14.04 (1.21)	
Hematocrit [%]		38–50
mean (sd)	42.28 (3.6)	
Erythrocytes [10^6^/μL]		4.25–6
mean (sd)	4.72 (0.4)	
Platelets [10^3^/μL]		150–400
median (IQR)	245.5 (212–287.8)	
Leukocytes [10^3^/μL]		4.5–11
median (IQR)	6.2 (5.3–7.3)	
Granulocytes [10^3^/μL]		1.6–7.6
median (IQR)	3.6 (3–4.4)	
Lymphocytes [10^3^/μL]		1–4.8
median (IQR)	1.8 (1.5–2.2)	
PT [s]		9.7–11.8
median (IQR)	11 (10–11)	
APTT [s]		22–29
median (IQR)	24 (23–25)	
	**n = 260**	**Reference value**
**Laboratory parameters**		
Fibrinogen [mg/dL]		170–420
median (IQR)	250 (227–290)	
D-Dimer [mg/L]		0–0.5
median (IQR)	0.2 (0–0.3)	
CRP [mg/dL]		0–0.5
median (IQR)	0.08 (0.04–0.18)	
Creatinin [mg/dL]		0.6–1.1
median (IQR)	0.86 (0.76–0.99)	
ALT [U/L]		0–50
median (IQR)	25 (17.75–33.25)	
Cholinesterase [U/L]		5859–13060
median (IQR)	9820 (8575–11125)	

**Table 2:** Distribution of blood groups of persons after COVID-19 compared to a historical collective.

	n = 426 (COVID-19)	n = 2434 (historical donor collective)	P.value
Blood group n (%)			
0	159 (37.3)	974 (40.1)	0.308
A	214 (50.2)	1055 (43.3)	0.523
B	32 (7.5)	295 (12.1)	0.0049
AB	16 (3.8)	110 (4.5)	0.608
NA	5 (1.2)		

### Laboratory parameters

The baseline laboratory parameters showed no pathological values except for slightly decreased hemoglobin levels in a few individuals (Table 1). The results of an extended laboratory analysis (fibrinogen, D-dimer, CRP, creatinine, ALT, cholinesterase) of a randomly selected subgroup of 260 persons showed a few abnormalities. Pathological values were measured for D-dimer (n = 21, range: 0.6–3.8 mg/L, ref. value: 0–0.5 mg/L), CRP (n = 17, range: 0.55–1.9 mg/dL, ref. value: 0–0.5 mg/dL), creatinine (n = 7, range: 1.2–1.3 mg/dL, ref. value: 0.6–1.1 mg/dL), ALT (n = 27, range: 51–145, ref. value: 0–50 U/L) and cholinesterase (n = 5, range: 1100–5430, ref. value: 5859–13060 U/L) (Table 1). These are not related to the duration of the disease or the time between the onset of symptoms and presentation at our clinic.

### Characteristics and duration of COVID-19

The course of the disease was very varied. However, only two persons in our cohort had to be hospitalized for one week (without ventilation) as a sign of a more severe course of the disease. Accordingly, we characterized the whole group as having a mild course. The median time between the onset of the first symptoms and the end of the disease was 12 days (IQR: 7–17), but varied from 0 to 45 days. The most common symptoms were fatigue (70.7%), cough (61.7%), olfactory and gustatory dysfunction (60.3%) as well as headache (57.2%) (Table 3). The symptoms that lasted longest over the period of the disease were cough and olfactory and gustatory dysfunction with a median of 7 days (IQR: 4–14 days) (Table 2, S2 Fig). The duration of fatigue (range: 0–60), as well as olfactory and gustatory dysfunction (range: 0–74) continued in some cases for a longer period of time after the other symptoms had subsided and the individuals considered themselves to be recovering.

**Table 3:** Characterization of COVID-19 in terms of symptoms and their duration.

	n = 426
**Duration of COVID-19 [days]**	
Median (IQR)	12 (7–17)
**Time between start of symptoms and antibody testing**	
Median (IQR)	50 (41–61)
**Symptoms n (%)**	
fever	204 (47.9)
cough	263 (61.7)
headache	244 (57.2)
sickness	50 (11.7)
olfactory & gustatory dysfunction	257 (60.3)
aching limbs	225 (52.8)
shivering	112 (26.3)
sore throat	149 (35)
shortness of breath	78 (18.3)
fatigue	301 (70.7)
diarrhoea	93 (21.8)
rhinitis	152 (35.7)
**Symptoms [days]**	
fever	
Median (IQR)	3 (2–5)
cough	
Median (IQR)	7 (4–14)
headache	
Median (IQR)	3 (2–5)
sickness	
Median (IQR)	3 (2–5)
olfactory & gustatory dysfunction	
Median (IQR)	7 (4–14)
aching limbs	
Median (IQR)	3 (2–6)
shivering	
Median (IQR)	2 (1–3)
sore throat	
Median (IQR)	4 (2–7)
shortness of breath	
Median (IQR)	4 (3–7.75)
fatigue	
Median (IQR)	7 (4–10)
diarrhoea	
Median (IQR)	2 (1–3)
rhinitis	
Median (IQR)	6 (3–10)

Among the 426 persons, 176 lived in a relationship / in a shared household. It was found that in 66 households both persons were infected and in 22 only one person was infected. Due to the small number of households with children, the children were not included in the evaluation.

### SARS-CoV-2 antibody and RT-PCR testing results

In 85.9% of the collective (n = 426), a throat swab had tested positive for SARS-CoV-2 by RT-PCR during the disease. Antibody testing revealed a positive result for specific IgG against SARS-CoV-2 in 82.4% and 73.9% for specific IgA of the participants (S3 Fig). In 15.2% of the individuals, only IgG and not IgA was detectable, and in 6.8% of the individuals only IgA and not IgG was detectable. 10.8% of the persons showed no antibodies against SARS-CoV-2 despite positive virus detection during the disease (Table 4). Of the 426 persons who were most likely to have had COVID-19, 24 were clinically asymptomatic. Nevertheless, IgG and/or IgA antibodies could be detected in 23 of them.

**Table 4:** Results of SARS-CoV-2 antibody and RT-PCR testing ("Anti-SARS-CoV-2 ELISA (IgG)","Anti-SARS-CoV-2 ELISA (IgA)" from EUROIMMUN AG, GenScript cPass™ SARS-CoV-2 Neutralization Antibody Detection Kit).

	n = 426
**Positive RT-PCR for SARS-CoV-2 n (%)**	366 (85.9)
**Positive antibodies against SARS-CoV-2 n (%)**	
IgG	351 (82.4)
IgA	315 (73.9)
IgG and IgA	286 (67.1)
only IgG	65 (15.3)
only IgA	29 (6.8)
**Positive RT-PCR and negative IgA and IgG antibody n (%)**	46 (10.8)
**Positive neutralization antibodies n (%)**	
** Group 1 (≥20%)**	43 (93.5)
** Group 2 (≥20%)**	44 (95.6)
** Group 3 (≥20%)**	46 (95.8)
** Group 1 (≥50%)**	14 (30.4)
** Group 2 (≥50%)**	36 (78.3)
** Group 3 (≥50%)**	46 (95.8)

The persons who showed no IgG, no IgA or neither IgG nor IgA, were compared with persons in whom the respective antibodies were detectable with regard to the duration of the disease and the time between the onset of symptoms and presentation in our clinic. Only the duration of the disease showed a significant difference (p.value = 0.032) between persons with no IgG antibodies and persons with IgG antibodies (Table 5). We also found that individuals with high levels of anti-SARS-CoV-2 IgG antibodies (ratio ≥6) had a significantly longer duration of illness than people with lower levels (ratio ≤2–1.1) (p.value<0.001) (Table 5).

**Table 5:** Association between results of antibody detection and time after start of symptoms or duration of COVID-19.

	Time between start of symptoms and antibody testing [days]	P.value	Duration of COVID-19 [days]	P.value
**Positive IgG antibodies against SARS-CoV-2**		0.189		0.032
Median (IQR)	51 (41–62) n = 329		12 (7–17) n = 351	
**Negative IgG antibodies against SARS-CoV-2 but positive RT-PCR**				
Median (IQR)	49 (42–56.5) n = 67		10 (5–15) n = 75	
**Positive IgA antibodies against SARS-CoV-2**		0.320		0.354
Median (IQR)	51 (42–62) n = 297		12 (7–17) n = 315	
**Negative IgA antibodies against SARS-CoV-2 but positive RT-PCR**				
Median (IQR)	51 (42–57) n = 78		11 (7–16) n = 89	
**Positive IgG or IgA antibodies against SARS-CoV-2**		0.877		0.516
Median (IQR)	51 (41–62) n = 358		12 (7–17) n = 380	
**Negative IgG and IgA antibodies against SARS-CoV-2 but positive RT-PCR**				
Median (IQR)	50 (42–57) n = 40		11 (7–15) n = 41	
**IgG antibodies against SARS-CoV-2 ratio ≥ 6**		0.671		<0.001
Median (IQR)	52 (43–62,5) n = 69		14 (10.25–20) n = 70	
**IgG antibodies against SARS-CoV-2 ratio >1.1 & ≤2**				
Median (IQR)	51 (42–62) n = 53		9 (6.75–16) n = 60	

In a subanalysis, we determined neutralizing antibodies in three groups of patients. The groups were defined based on IgG antibody results (group 1, n = 46, ratio: >1.1 - ≤2; group 2, n = 46, ratio: >2 - ≤4; group 3, n = 48, ratio: >4–10). When using the manufacturer’s cut-off for a positive result (≥20%), we find in all 3 groups that more than 90% of donors show a positive result. If the cut-off according to Rein et al. [18] is adjusted, it can be seen that in group 1 only 30%, in group 2 78% and in group 3 still 96% of the donors are positive (S4 Fig). Based on this result, a correlation was calculated which showed a rho of 0.720 and a p.value of <0.001.

### Donation of CP

Based on the laboratory tests and anamnesis conducted, a total of 319 out of 485 persons were suitable for CP donation. Only individuals with a negative COVID-19 swab result, as well as IgG antibody ratios of more than 1.1, were qualified as CP donors. By August 7^th^, 2020 we had collected 918 donations from 281 donors.

## Discussion

In this study, we included persons between April 1^st^, 2020 and July 16^th^, 2020 who had overcome COVID-19 and had volunteered to donate CP.

Based on the subjective self-assessment of the donors regarding their ability to donate CP, it was a cohort describing the part of the population that is fundamentally healthy (exclusion of cancer, chronic disease,…). The potential donors were examined with regard to their disease course, laboratory parameters and antibody status.

Of the 485 people who presented to our clinic for an initial examination, 426 people can be assumed to have recovered from COVID-19 based on the results of the throat swab and antibody test. Among this collective of 426 persons, there were some who were not detected by RT-PCR during the disease (n = 60). This was due to the fact that some health authorities only tested one person from each household or travel group. One person tested positive from these groups was then sufficient to establish quarantine.

Except for two persons, each of whom had to be hospitalized for one week (without ventilation), the course of the disease was mild and lasted 12 days in the median. This corresponds to other descriptions of mild COVID-19 courses [19]. In the persons who reported a duration of the disease of >21 days, the longest remaining symptoms were coughing, olfactory & gustatory dysfunction and fatigue, with other symptoms otherwise subsiding.

The laboratory analyses of the parameters of hematology, hemostaseology and clinical chemistry did not show any particular abnormalities within the framework of the tests performed at our facility. The pathological values were only slightly increased or decreased and were not further evaluated statistically because of the small number of pathological parameters per se.

There was only a significant difference in the blood group distribution for blood group B compared to the usual donor pool at our clinic (Table 2). Wu et al. reported that people with blood group A have a higher risk of infection with SARS-CoV-2 [20]. This observation could not be made in our collective, which could be due to the fact that only people with a mild course of the disease were included in our study. The role of the blood group is still under discussion, although it is currently assumed that it has no significant influence on the severity of the disease or the likelihood of death [21, 22].

In 67% of the spouses or households examined, both persons had recovered from COVID-19. Whether the persons in one household were infected at the same time, and/or whether the transmission took place inside the household cannot be clarified here. The data on household transmissions described in the literature show values of 15,6% [23] or 23% [24]. This is much lower compared to our collective. Since many couples were on holiday together (especially in Ischgl, Austria, one of the first hotspots in Europe), it can be postulated that most couples were infected at the same time and that the transmission did not take place in the joint household.

Interestingly, antibodies to SARS-CoV-2 could not be detected in all individuals, although they had a positive throat swab for SARS-CoV-2 during the disease. An association with certain symptoms or the time between illness and presentation in our clinic could not be found in these persons (Table 5), since 23 of the 24 persons (96%) with asymptomatic courses of the disease also produced antibodies.

As Long et al. were able to show, the titer of the antibodies drops already just a few weeks after the disease [25]. The people who no longer showed any detectable antibodies during the examination in our clinic had possibly only produced a small amount of antibodies during the illness [26], which then quickly fell below the detection limit. A possible reason for this could be that, for example, cross-reactive immunity [27] or the complement system [28] was able to eliminate most of the virus and thus only a small part of the virus remained to induce antibody formation [29]. What this means for the immunity of these persons is still unclear. Confirmed secondary infections have not yet been published, which is why a certain immunity can be suspected even in persons without detectable antibodies. This observation could also explain the significant difference in disease duration between people with detectable IgG antibodies and people without IgG antibodies against SARS-CoV-2 (Table 5). Based on the work of Long et al., it would be interesting to observe how antibody titers behave in the further follow-up of the disease and whether they have a direct influence on immunity against a renewed SARS-CoV-2 infection [25].

A critical factor for immunity against SARS-CoV-2 infection are neutralizing antibodies. The assays used in this study to determine IgG/IgA antibodies against SARS-CoV-2 and to determine neutralizing antibodies against SARS-CoV-2 are semiquantitative and qualitative methods, respectively. Nevertheless, we were able to show a correlation between these two assays (rho: 0.720, p.value <0.001). Furthermore, we could show that individuals with a high ratio (≥6) in the antibody assay (IgG) had a significantly longer duration of COVID-19 than individuals with a low ratio (≤2 - >1.1) (Table 5). This suggests that the immune systems of individuals with a longer disease course also produce more antibodies against SARS-CoV-2. Such correlations have also been shown in other studies [30–32].

Overall, this work shows that it is also important to investigate the weaker courses in collectives with regard to hematology, hemostaseology and clinical chemistry. Here, our study could not detect any abnormalities a few weeks after COVID-19. Furthermore, these collectives are essential to clarify questions of long-term immunity. Since it was also shown in our study that the antibodies were no longer detectable in some of the donors, follow-up examinations of the cellular and non-cellular immune response would be important to answer questions about the duration and type of immunity.

### Limitations

There are some limitations to this study. The people included in the study felt themselves to be eligible to donate. Thus, this is not a random sample collective that was selected independently of the underlying diseases.

Furthermore, only persons between 18 and 70 years of age were enrolled. The persons included came from a small catchment area of about 150 km and were persons with a mild COVID-19 course. Furthermore, there were only 88 households with 2 or more persons in the study collective, so that a statement about the infection rate in households is only possible to a very limited extent.

## Supporting information

S1 Fig Time parameters related to COVID-19. (EPS)

S2 Fig Duration of COVID-19 and each symptom. (EPS)

S3 Fig Results of SARS-CoV-2 antibody testing. (EPS)

S4 Fig Neutralization antibody detection. (EPS)

S1 Table Raw data. (XLSX)
